# Nestedness theory suggests wetland fragments with large areas and macrophyte diversity benefit waterbirds

**DOI:** 10.1002/ece3.8009

**Published:** 2021-08-16

**Authors:** Rongxing Wang, Xiaojun Yang

**Affiliations:** ^1^ State Key Laboratory of Genetic Resources and Evolution Kunming Institute of Zoology Chinese Academy of Sciences Kunming China; ^2^ Institute of Eastern‐Himalaya Biodiversity Research Dali University Dali China

**Keywords:** aquatic vegetation, artificial wetlands, Lake Dianchi, nestedness, urbanization, waterbird conservation

## Abstract

Many artificial wetland constructions are currently underway worldwide to compensate for the degradation of natural wetland systems. Researchers face the responsibility of proposing wetland management and species protection strategies to ensure that constructed wetlands positively impact waterbird diversity. Nestedness is a commonly occurring pattern for biotas in fragmented habitats with important implications for conservation; however, only a few studies have focused on seasonal waterbird communities in current artificial wetlands. In this study, we used the nestedness theory for analyzing the annual and seasonal community structures of waterbirds in artificial wetlands at Lake Dianchi (China) to suggest artificial wetland management and waterbird conservation strategies. We carried out three waterbird surveys per month for one year to observe the annual, spring, summer, autumn, and winter waterbird assemblages in 27 lakeside artificial wetland fragments. We used the NeD program to quantify nestedness patterns of waterbirds at the annual and seasonal levels. We also determined Spearman partial correlations to examine the associations of nestedness rank and habitat variables to explore the factors underlying nestedness patterns. We found that annual and all four seasonal waterbird compositions were nested, and selective extinction and habitat nestedness were the main factors governing nestedness. Further, selective colonization was the key driver of nestedness in autumn and winter waterbirds. We suggest that the area of wetland fragments should be as large as possible and that habitat heterogeneity should be maximized to fulfill the conservation needs of different seasonal waterbirds. Furthermore, we suggest that future studies should focus on the least area criterion and that vegetation management of artificial wetland construction should be based on the notion of sustainable development for humans and wildlife.

## Graphical abstract

We use the nestedness theory to analyze the community structures across different seasonal waterbirds in artificial wetlands at Lake Dianchi and explore their underlying mechanisms in order to suggest artificial wetland management and waterbird conservation strategies. We found that annual and all four seasonal waterbird compositions were nested, and selective extinction and habitat nestedness were the main factors governing the nestedness. We suggest that the area of fragmented wetlands should be as large as possible and that habitat heterogeneity should be as great as possible to fulfill the conservation needs of different seasonal waterbirds.

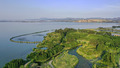

## 1 INTRODUCTION

As a combined result of climate change and anthropogenic activities, global wetlands have been severely degraded over the past century, and their ecosystem services and functions have experienced significant degeneration (Amano et al., 2018, 2020). In addition, waterbird populations that depend on wetlands for survival have markedly declined (Amano et al., 2018; Hu et al., 2017). The protection of wetland ecosystems and biodiversity has become a global concern (Ramsar Convention Secretariat, 2016). To reduce the degradation of natural wetlands, numerous wetland restoration and artificial wetland construction projects are currently underway globally (Darrah et al., 2019). These newly constructed wetlands fulfill multiple functions, such as contamination abatement and human recreation, and oxidation ponds are the most important construction form (Zhao et al., 2020). Waterbirds are vital constituents of wetland ecosystems and positively impact wetland health (Amat and Green, 2010). The protection of global waterbirds depends on effective governance (Amano et al., 2018). The main problem faced by ecosystem designers is constructing the most suitable artificial wetlands for the survival of waterbirds (Almeida et al., 2020). Ensuring that the constructed wetlands promote waterbird diversity and designing wetland management and waterbird protection measures are important tasks entrusted to scientists and managers (Giosa et al., 2018).

Community composition is one of the three primary descriptors (species richness, abundance, and composition) of community structure. Composition plays an essential role in studying the relationships between species and their environments (Worthen, 1996), and it is a factor that must be considered in the design of species protection and habitat management projects (Marini et al., 2019). Community composition is affected by many factors, including intrinsic factors of community species (such as intrinsic growth rate) and extrinsic factors (such as the natural environment and anthropogenic disturbance; Darlington, 1957). Currently, nestedness analyses are promoted as key investigative tools for identifying the mechanisms that potentially structure a community (Ulrich et al., 2017; Worthen, 1996). The nested pattern was described for island habitats by Darlington (1957), who stated that the species composition of a small island or fragment tends to be a subset of an adjacent larger island or fragment (Cutler, 1994). Further, the species comprising a depauperate insular biota consist of a proper subset of those in richer biotas (Patterson, 1987). The system is perfectly nested if all species in the small island are also found in the adjacent larger island; however, this perfectly nested pattern rarely occurs in nature (Wright et al., 1998). Nestedness, to some extent, is one of the most frequently occurring patterns for biotas in the island landscape (Wang et al., 2013; Wright et al., 1998), including birds (Fernández‐Juricic and Jokimäki, 2001; Murgui, 2010; Wang et al., 2013), fish (Fernández‐Juricic and Jokimäki, 2001), insects (Fernández‐Juricic, 2002; Fernández‐Juricic and Jokimäki, 2001; Xu et al., 2017), plants (Platt and Lill, 2006), mammals (Chen et al., 2019), reptiles (Wang et al., 2010), and macroinvertebrates (Florencio et al., 2011; Williams‐Subiza et al., 2020).

Moreover, the mechanisms underlying nested patterns among various biotas differ (Fischer and Lindenmayer, 2005; Wang et al., 2010). In addition to the insular habitat, the nested pattern is commonly present in land‐bridge island and landscape fragment habitats (Fischer and Lindenmayer, 2005; Patterson, 1987). Ulrich et al. (2009) summarized several mechanisms that have been proposed to account for nestedness. Among them, five have been frequently used to explain the nestedness phenomenon: selective colonization, selective extinction, habitat nestedness, passive sampling, and anthropogenic disturbance (Wang et al., 2013). The selective colonization hypothesis predicts that the species with the strongest dispersal ability will occupy more habitats than the species with the weakest dispersal capacity and that fragment isolation will create nested subsets of species through dispersal limitation (Cook and Quinn, 1995; Patterson, 1987; Wright et al., 1998). The selective extinction hypothesis predicts that area is the key factor explaining species nestedness; this is because species with larger area requirements exhibit greater extinction risks, as they will not appear in small habitat areas (Darlington, 1957; Patterson, 1987, 1990). The habitat nestedness hypothesis suggests that habitat nestedness will create corresponding subsets of species assemblages (Cook and Quinn, 1995). The passive sampling hypothesis considers that the detection probabilities of different species are related to their dominance; for example, in a given habitat area, common species are more likely to be observed than rare species (Cutler, 1994; Schouten et al., 2007). Finally, the anthropogenic disturbance hypothesis suggests that anthropogenic disturbance can promote nestedness (Fernández‐Juricic, 2002). These hypotheses complement each other and emphasize different aspects of nestedness. In most cases, they act together to form an observed nested pattern (Ulrich et al., 2009).

The causes of nested subsets are complex and differ between biotas or habitats within landscape fragments (Wang et al., 2010). Currently, nested analyses on urban avian assemblages have mainly focused on urban parks or woodlots and most of the study subjects were forest birds, especially resident birds and summer visitors (Li et al., 2019; Wang, Chen, et al., 2018). The mechanisms underlying nestedness may vary across different seasons at a given location because the habitat requirements, territories, and population parameters of migratory species display temporal variations (Fernández‐Juricic, 2002; Murgui, 2010). However, to date, only a few studies have focused on the seasonal patterns of nestedness (Murgui, 2010; Wang et al., 2013), and a limited number of studies have examined whether the mechanisms underlying nestedness vary among seasonal waterbirds in urban lakeside wetland fragments (Benassi et al., 2007). Therefore, it is necessary to study nestedness patterns for developing measures that protect seasonal waterbirds.

**FIGURE 1:**
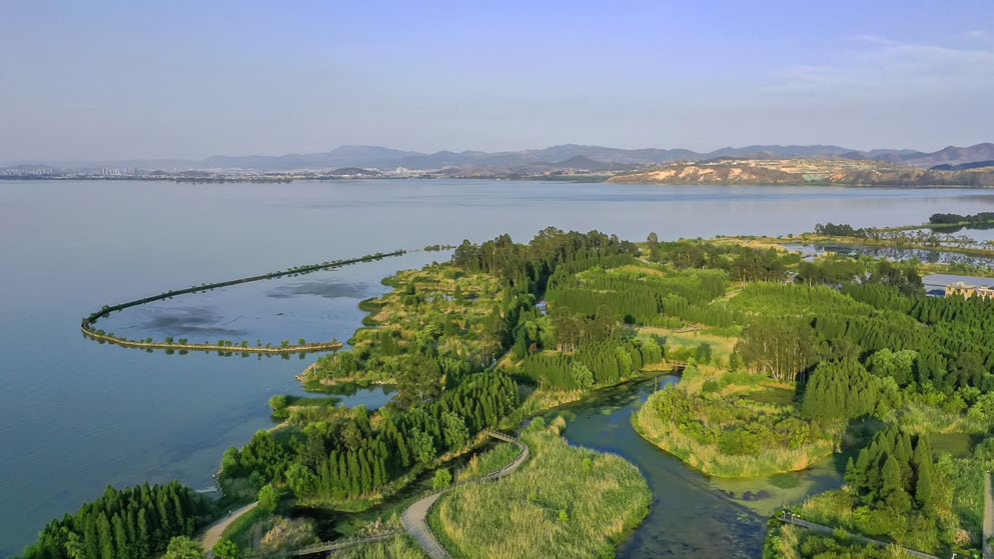
One of the lakeside artificial wetland fragments around Lake Dianchi, China

Lake Dianchi (hereafter Dianchi) is one of the most eutrophicated lakes in China (Zhang, Luo, et al., 2020). Its lakeside artificial wetland fragments have been constructed for water purification and ecotourism (Figure 1). In this study, we observed different seasonal waterbird distributions across 27 lakeside wetland fragments around Dianchi in relation to the following questions. (a) Do the distributions of annual and seasonal waterbird assemblages follow a nested pattern in the studied wetlands? (b) Do the mechanisms underlying nestedness vary among different seasonal waterbirds? (c) Can these results be applied to the conservation management of urban waterbird assemblages?

## 2 MATERIALS AND METHODS

### 2.1 Study area

**FIGURE 2:**
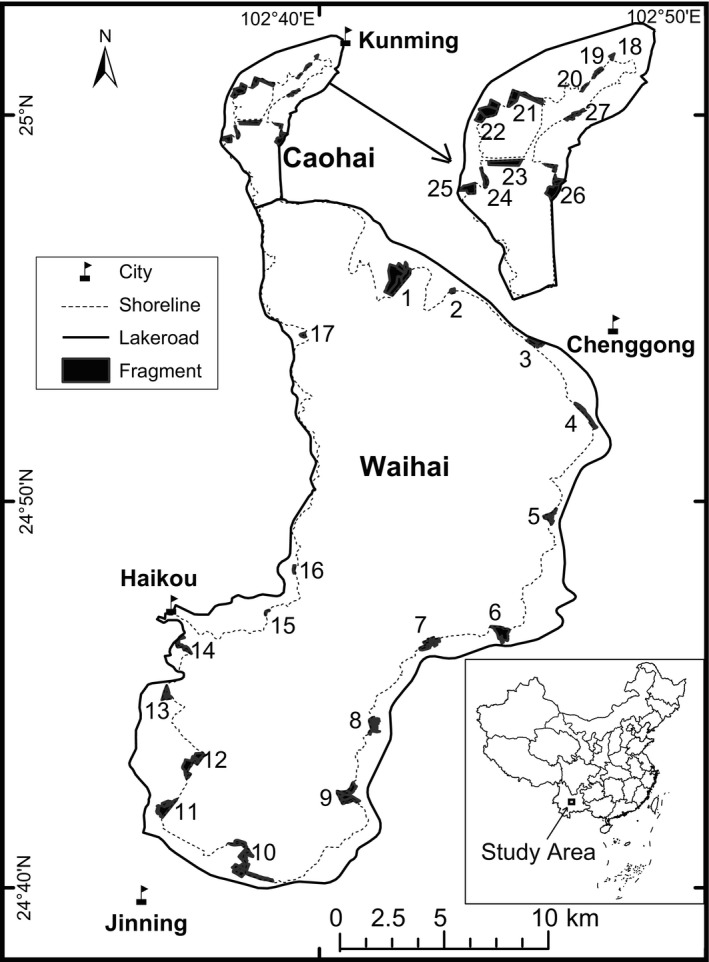
Locations of the 27 lakeside wetland fragments around Lake Dianchi, China. The upper right inset shows the locations of fragments in the Caohai area, and the bottom right shows the location of Lake Dianchi in China

Dianchi, located in southwest China (N24°40′–25°02′, E102°37′–102°48′), is the sixth‐largest freshwater lake in China and the largest on the Yunnan–Guizhou Plateau; it exhibits an area of 308.6 km^2^ and an average elevation of 1,888 m a.s.l. (Figure 2). It is an ancient tectonic lake separated into two parts by an artificial causeway. The northern part, Caohai, has a total water area of 10.7 km² and a mean water depth of 2.5 m. The southern part, Waihai, has a total water area of 297.9 km² and a mean water depth of 4.3 m (Jin et al., 2006). The climate is subtropical, with a mean temperature of 14.7℃, average annual precipitation of 797–1,007 mm, and 227 frost‐free days per year (Yang, Zhou, et al., 2010). The lake is nearly semicircular, and the shoreline is approximately 150 km in length. The lake body is 40.4 km long with an average width of 7.0 km. More than 20 streams flow into Dianchi from the northern, eastern, and southern directions, and their broad, flat, alluvial fans provide agricultural livelihoods for people living in Kunming Municipality (Yang et al., 2010). Situated within the heavily urbanized Kunming Municipality, Dianchi has become increasingly eutrophic since the 1980s because its self‐purification ability has been unable to match the massive discharge of municipal and industrial sewage into the water. In recent years, many projects have been initiated to control external nutrient loads. As one of these projects, artificial wetlands have been constructed by removing farmlands, factories, and residential buildings from the lakeside and relocating them elsewhere. Macrophytes have been planted in these artificial wetlands to remove the pollutants (Wang et al., 2012, 2016).

### 2.2 Sampling method

#### 2.2.1 Landscape fragments

We sampled 27 lakeside wetland fragments (hereafter fragments) around Dianchi (Figure 2). Each fragment was composed of several patches, whose boundaries were formed by roads, brooks, and belts of wood or shrub vegetation. The features of each fragment are shown in Table 1 and Table S1. We used the geometric center point to measure the distances between fragments, and the minimum distance was 300 m (Table S2). The width of the nonwetland belts between any two fragments, such as forest agricultural land, was more than 100 m. We previously performed a set of Mantel tests to explore whether waterbird composition and habitat variables are spatially autocorrelated (Legendre et al., 2015). The area of each fragment and distance between fragments were calculated using ArcGIS 9.0 (ESRI Inc.), and the Mantel test was performed using the package “‘Vegan” (Oksanen et al., 2020) in R version 4.0.1 (R Core Team, 2017).

**TABLE 1:** Characteristics of 27 lakeside wetland fragments around Lake Dianchi, China

Fragment code	Area (hectares)	Shannon ‐Wiener Diversity Index of habitat	Isolation 1 (km)	Isolation 2 (km, for annual, spring, summer, autumn birds)	Isolation 2 (km, for winter birds)	Human disturbance degree	Observed richness (*n*)					Nestedness matrix rank after rearranging by NeD				
							Annual	Spring	Summer	Autumn	Winter	Annual	Spring	Summer	Autumn	Winter
S01	119.90	1.05	2.60	28.91	17.42	1	31	11	15	17	12	26	24	25	25	22
S02	3.81	0.39	2.60	28.66	16.06	4	7	4	4	3	4	9	9	9	6	11
S03	19.98	0.69	4.39	28.41	14.22	2	15	5	9	6	12	20	13	21	15	23
S04	14.57	1.29	4.39	25.98	10.89	1	19	7	9	9	16	21	20	22	20	26
S05	16.13	0.68	6.58	21.83	6.58	4	7	5	6	4	7	10	14	15	9	18
S06	39.62	0.28	2.70	15.56	0.00	1	22	8	8	11	17	23	22	19	22	27
S07	16.67	1.03	2.70	13.78	2.70	3	28	15	16	16	9	25	25	26	24	19
S08	29.69	1.26	4.18	9.09	6.56	2	22	15	12	11	15	24	26	24	23	25
S09	51.41	1.05	5.49	5.49	10.24	1	14	8	10	5	4	18	23	23	13	10
S10	58.28	1.37	5.49	0.00	15.56	4	36	20	30	21	10	27	27	27	27	21
s11	39.24	1.12	4.53	4.53	16.47	1	11	4	5	7	11	16	10	13	16	20
S12	48.83	1.24	2.33	5.46	14.50	2	13	4	8	8	6	17	11	20	17	15
S13	16.38	0.99	3.16	8.62	14.51	1	15	5	6	9	14	19	15	16	21	24
S14	19.09	0.35	3.08	11.28	13.50	2	2	1	1	1	2	1	2	1	1	5
S15	2.08	0.00	3.83	12.06	9.85	1	7	4	2	4	5	7	12	5	10	13
S16	3.43	0.00	11.02	14.20	9.11	4	5	3	1	3	2	4	6	2	7	4
S17	4.62	0.00	11.02	25.18	16.12	3	20	2	2	17	5	22	4	6	26	14
S18	1.44	0.00	14.02	39.18	28.91	4	3	0	1	2	2	2	1	3	4	6
S19	3.35	0.00	0.30	38.15	28.05	1	6	3	5	1	2	5	7	14	2	3
S20	1.47	0.00	1.70	38.13	28.13	2	9	2	1	4	4	14	5	4	11	9
S21	17.52	0.68	1.41	38.03	28.66	1	8	6	4	5	2	12	18	10	14	7
S22	24.66	1.02	1.41	37.00	28.08	2	8	5	4	8	7	11	16	11	18	16
S23	14.80	0.00	1.12	36.01	26.96	3	7	5	4	2	0	6	17	12	5	2
S24	11.17	0.19	1.17	35.00	26.28	1	10	3	6	3	7	15	8	17	8	17
S25	10.58	0.00	0.40	35.00	26.44	2	9	7	2	8	5	13	21	7	19	12
S26	5.34	0.72	2.30	35.07	25.56	2	4	1	2	1	0	3	3	8	3	1
S27	7.55	0.61	1.72	37.11	27.26	2	7	6	6	4	3	8	19	18	12	8

Note

Isolation 1 is the distance of each fragment to the adjacent larger fragment; Isolation 2 is the distance of each fragment to the annual, spring, summer, autumn pool fragment (Site 12) and to the winter pool fragment (Site 1); the anthropogenic disturbances were classified into four degrees: 1, light disturbance (pedestrian or angling); 2, moderate disturbance (motorcycle riding or fishing); 3, severe disturbance (car driving or pasturing); and 4, extreme disturbance (wetland park).

#### 2.2.2 Classification of habitat type

According to a previous study (Wang et al., 2016), the habitat of each patch was classified into seven types: pond, mudflat, high emerging plant, low emerging plant, high floating plant, low floating plant, and mixed vegetation. The habitat area in each patch was represented as the patch area. The patch area was stable across different seasons because the patches were not subjected to human disturbance during the entire survey period. We summed the area of each habitat in the fragment and calculated the Shannon–Wiener diversity index (SHDI). SHDI was used as the indicator of the habitat diversity of each fragment (Table S1).

#### 2.2.3 Classification of anthropogenic disturbance

Based on the road type, major anthropogenic activities, or function of the fragment, we classified the anthropogenic disturbance into four levels: light, moderate, severe, and extreme (Table 2). The highest level of severity was used as the human disturbance level for each fragment.

**TABLE 2:** Classification of anthropogenic disturbance

Disturbance type	Severity status	Severity level
Road type		
Car driving	Severe	4
Motorcycle riding	Moderate	3
Pedestrian	Light	2
Fragment's function		
Angling	Light	1
Fishing	Moderate	2
Pasturing	Severe	3
Park	Extreme	4

#### 2.2.4 Seasonal classification and waterbird count

Bird populations are dynamic, and nestedness patterns may change with seasons (Murgui, 2010; Wang and Yang, 2020). We used the annual and seasonal patterns to discuss nestedness, which would also have more significance in practical management (de la Hera, 2019). According to the phenology and seasonal classification method widely used in China, we established seasons as spring (March–May), summer (June–August), autumn (September–November), and winter (December–February).

We optimized and fixed the survey routes and adopted the spot‐map census method to mark the species and individuals on prepared maps based on patches (Bibby et al., 2000). Binoculars (Olympus 10 × 42 EX WP I) and telescopes (Carl Zeiss DiaScope 85T*FL) were used to observe waterbirds. We carried out waterbird surveys three times each month (early, middle, and late) from March 2013 to February 2014. Each survey was conducted on three consecutive days from dawn to dusk. We divided all fragments into three sections according to the total survey time for a single survey and began the survey (in the morning) from each section during the same month to reduce bias. The time windows were advanced or delayed by one or two days if weather conditions were adverse (e.g., rain, heavy fog, snow, or gales; Bibby et al., 2000; Conway, 2011).

For each fragment, we included the surveyed individuals as the abundance of each species. We used the maximum abundance of each species as the monthly population since each fragment was surveyed at morning, noon, and afternoon in each month. We summed the abundance calculated for three months as the corresponding seasonal abundance for each species and the abundance determined for all 12 months as the annual abundance. We used the Mao Tao estimator to determine our sampling adequacy and estimated species richness (Colwell et al., 2012). The extrapolated bird species richness of each fragment was calculated using the common nonparametric test Chao1 (Chao et al., 2014). The analyses were performed using the online program iNEXT (Hsieh et al., 2016; http://chao.stat.nthu.edu.tw/wordpress/software_download/inext‐online).

### 2.3 Quantification of nestedness

We used the online program NeD (Nestedness for Dummies, http://ecosoft.alwaysdata.net/doc/), which was supplied by Strona and Fattorini (2014), to count the nestedness metric for determining whether the bird communities and habitat type among the fragments showed nested patterns. We chose CE null models (proportional row totals and proportional column totals) to compute *Z* values; a value >1.64 indicated significance at *p* = .05 (Strona et al., 2014). The CE null model held a higher conservativeness and ecological realism, and it is considered a preferable null model algorithm for nestedness analysis (Strona and Fattorini, 2014). In addition, we used the NODF program to count the weighted NODF (WNODF) metrics, which were based on the species and its abundance (incidence). We used all three null models (*aa*, *ss*, and *rc*) to test the significance of nestedness among fragments (Almeida‐Neto and Ulrich, 2011). *p* < .05 was considered significant.

### 2.4 Determinants of nestedness

#### 2.4.1 Passive sampling tests

We used the random placement model to test the passive sampling hypothesis. Using the model, the number of species *S*_(_ *_α_*_)_ in a given fragment *r* depends on its relative area *α* (*α* = *a_r_*/*A*, where *a_r_* is the area of fragment *r*, and *A* is the total area of all fragments). The abundance of species *i* was represented by *n_i_*. For *i* = 1,…, *S*, the overall abundance *n*_1_, *n*_2_, … , *n_s_* of *S* species represented in collection C (Coleman, 1981; Coleman et al., 1982): *S*_(_ *_α_*_)_ = $S‐\sum_{i=1}^{S}{\left(1‐\alpha \right)}^{n_{i}}$. The variance *σ* ^2^ of *S*_(_ *_α_*_)_ is determined as $\sigma_{\left(\alpha \right)}^{2}$ = $\sum_{i=1}^{S}{\left(1‐\alpha \right)}^{n_{i}}‐\sum_{i=1}^{S}{\left(1‐\alpha \right)}^{2n_{i}}.$ The random distribution hypothesis should be rejected if more than one third of the points lie outside one standard deviation of the expected curve (Coleman, 1981; Coleman et al., 1982).

#### 2.4.2 Habitat variable tests

For each fragment, we selected several habitat variables that are commonly considered to influence species nestedness: area, isolation, habitat diversity, and human disturbance (Chen et al., 2020; Wang et al., 2013). Measures of isolation included distance to the nearest larger fragment (Isolation 1) and distance to the fragment, which held the highest species richness (species pool, Isolation 2; Wang et al., 2013; Tan et al., 2020). We used principal component analysis (PCA) to describe the corrections among the above habitat variables under the rank orders of fragments after developing the species‐by‐site matrix using NeD. We also conducted partial Spearman rank correlations to examine the associations between variables and nestedness rank to test the selective extinction, selective colonization, habitat nestedness, and anthropogenic disturbance hypotheses (Ding et al., 2013; Schouten et al., 2007; Wang et al., 2010). The PCAs were performed using the package “Vegan” (Oksanen et al., 2020) in R version 4.0.1 (R Core Team, 2017).

#### 2.4.3 Ecological trait tests

We collected data on six life‐history ecological traits associated with bird colonization and extinction rate: body size, clutch size, geographic range size, dispersal ratio, occupied habitat (habitat specificity), and minimum area requirement (Table 2; Tan et al., 2020). The information regarding the first four traits was acquired from data published by Wang et al. (2018). The occupied habitat was factored in as the number of habitat types used by a given species around a year in Dianchi, and the minimum area requirement was accounted for as the minimum fragment area occupied by a given species (Wang et al., 2010). We assumed that the species with larger body and/or clutch sizes, or minimum area requirements, needed larger habitat areas and were more at risk of extinction in smaller fragments. In contrast, species with higher geographic range sizes and dispersal ratios and more occupied habitat were regarded as generalists and could be observed in more fragments. We conducted a partial Spearman rank correlation of species nestedness rank and species ecological traits to test the selective extinction and selective colonization hypotheses. The partial Spearman tests were performed in R version 4.0.1 (R Core Team, 2017).

## 3 RESULTS

### 3.1 General sampling results

**FIGURE 3:**
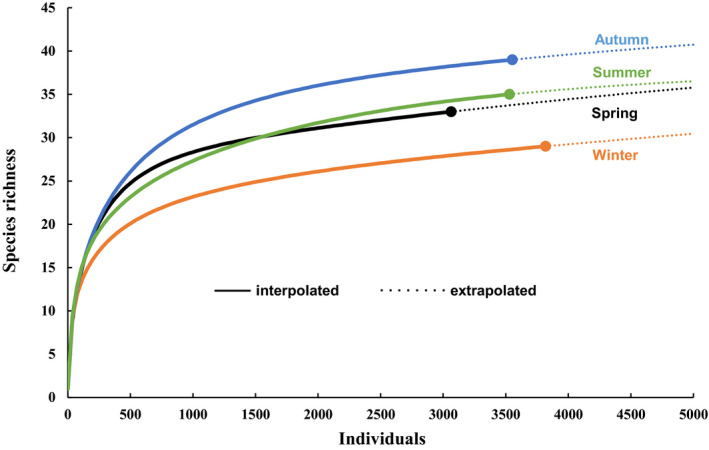
Species accumulation curve for different seasonal waterbirds in 27 lakeside wetland fragments around Lake Dianchi, China

In total, 53 waterbird species were recorded across the entire surveyed period (annual) in the 27 fragments. In addition, 33, 34, 40, and 28 species were recorded in spring, summer, autumn, and winter, respectively (Table 3). According to the expected species richness, the survey completeness for the 27 fragments was very high, ranging from 62.5% to 100% for the annual and individual seasonal surveys (Table S1). The abundance of each species in the annual and seasonal surveys is shown in Table S3. The species accumulation curve approached an asymptote for each season (Figure 3), indicating that the survey effect was sufficient to support subsequent analyses.

**TABLE 3:** Annual and seasonal species and accumulated individuals in the 27 lakeside fragments around Lake Dianchi between March 2013 and February 2014

Species	Body size (mm)	Clutch size (*n*)	Occupied habitat (*n*)	Geographic range size (km^2^)	Dispersal ratio (dp)	Area requirement (ha)	Accumulated individuals					Nested matrix rank after rearranging by NeD				
							Annual	Spring	Summer	Autumn	Winter	Annual	Spring	Summer	Autumn	Winter
*Egretta garzetta*	596.5	2.5	7	295.09	35.1	1.47	1,736	214	678	453	391	1	2	1	3	1
*Gallinula chloropus*	190	8	6	961.58	12.18	1.44	2,829	887	781	768	393	2	1	2	2	2
*Amaurornis phoenicurus*	302	6	5	526.51	17.27	1.44	138	44	48	20	26	3	4	3	5	7
*Ardeola bacchus*	262.15	3	5	908.62	32.01	1.44	458	76	15	269	98	4	5	9	1	4
*Podiceps ruficollis*	158.15	5.5	4	961.58	18.92	1.47	1,289	347	326	317	299	5	3	5	4	3
*Ixobrychus sinensis*	331.5	7	4	606.69	18.91	1.47	54	3	41	8	2	6	27	4	9	18
*Larus ridibundus*	386.75	3	2	961.58	25.68	1.58	1,190	109	7	118	956	7	8	20	15	5
*Bubulcus ibis*	509.75	6	4	955.92	33.99	1.58	690	232	81	155	222	8	6	10	7	9
*Porzana fusca*	115.75	7	4	600.05	15.86	1.47	55	16	32	7		9	7	8	20	
*Egretta intermedia*	666.5	2	4	291.12	39.68	3.35	81	10	57	14		10	15	6	13	
*Ardea cinerea*	888	5	3	961.58	38.32	1.47	170	7	29	47	87	11	13	15	12	6
*Tringa glareola*	110	2	3	961.58	31.06	4.62	143	14	98	23	8	12	12	7	8	12
*Capella gallinago*	171.5	2	4	961.58	15.08	4.62	130	16	3	43	68	13	9	17	6	8
*Tringa hypoleucos*	189.15	2.5	3	961.58	19.52	4.62	30		8	10	12	14		13	14	10
*Charadrius dubius*	168	3.5	3	961.58	32.53	4.62	63	9	47		7	15	19	12		11
*Vanellus cinereus*	321	2	4	676.36	35.57	2.8	281	23		234	24	16	11		10	13
*Ixobrychus cinnamomeus*	329.5	2.5	3	360.9	17.59	1.47	24	8	11	5		17	10	11	26	
*Tringa ochropus*	132	3.5	3	961.58	31.12	4.62	40	23	8	7	2	18	14	34	21	20
*Nycticorax nycticorax*	515	2	3	821.36	31.92	1.58	44			42	2	19			11	22
*Charadrius alexandrinus*	161.5	2	3	873.52	31.08	4.62	16		1	9	6	20		30	27	14
*Capella stenura*	151.15	2	3	961.58	17.31	4.62	11		3	3	5	21		25	23	15
*Pluvialis fulva*	121.15	2.5	3	961.58	31.86	4.62	7		3	4		22		19	19	
*Fulica atra*	391	9	3	961.58	12.86	24.66	118	29	1	11	77	23	30	31	30	16
*Himantopus himantopus*	353.75	2	3	961.58	21.02	4.62	224	10	187	27		24	29	22	16	
*Charadrius placidus*	110.75	3.5	3	796.58	32.05	4.62	10		7	3		25		14	24	
*Charadrius mongolus*	188.5	3	3	612.69	33.11	4.62	11	7		4		26	17		25	
*Anas crecca*	388.5	9.5	3	961.58	16.18	14.57	52	1		10	41	27	26		18	27
*Anas poecilorhyncha*	570.5	9.5	3	961.58	15.91	16.13	58	9	32	16	1	28	33	33	31	21
*Calidris ferruginea*	110	2	2	905.97	31.51	4.62	18	5	6	7		29	24	28	35	
*Anas strepera*	299.5	10	2	961.58	17.18	29.69	146	19			127	30	31			17
*Calidris subminuta*	153.15	2	3	961.58	18.81	4.62	18		11	7		31		26	17	
*Calidris alpina*	195.5	2	2	631.37	19.2	4.62	2			2		32			28	
*Tadorna ferruginea*	592	9	2	959.02	31.57	39.62	21			5	16	33			39	23
*Numenius phaeopus*	217.15	2	1	887.38	31.11	16.67	6		1	5		34		24	22	
*Tringa erythropus*	193	2	3	961.58	30.35	58.28	3			3		35			29	
*Glareola maldivarum*	131.5	3	2	658.72	21.21	16.67	106	42	64			36	16	18		
*Tringa totanus*	170	2	3	860.71	30.81	58.28	7	4	3			37	28	21		
*Porzana pusilla*	172.15	7.5	3	838.81	12.62	51.41	4	1	2	1		38	22	16	34	
*Anas platyrhynchos*	523.75	9	3	961.58	16.78	16.67	5	4			1	39	18			26
*Hydrophasianus chirurgus*	225	2	3	191.11	37.1	39.62	4	1			3	40	25			19
*Calidris minuta*	140	3.5	2		32.97	58.28	4	1	3			41	23	27		
*Ixobrychus eurhythmus*	335.5	2	1	623.35	17.68	39.62	2		1	1		42		23	32	
*Larus brunnicephalus*	221	3	2	629.57	21.03	1.58	13	12			1	43	20			28
*Botaurus stellaris*	676.75	5	2	771.69	31.6	11.17	1				1	44				24
*Arenaria interpres*	115.15	2	2	857.37	31.51	119.9	1			1		45			38	
*Calidris ruficollis*	156.15	2	2	961.58	31.5	4.62	2			2		46			36	
*Recurvirostra avosetta*	217.75	2	1	959.02	31.78	4.62	1			1		47			37	
*Ardea purpurea*	901.5	2.5	2	606.69	36.2	58.28	1			1		48			40	
*Anas acuta*	567.5	8.5	2	961.58	18.15	16.67	2			2		49			33	
*Rostratula benghalensis*	150.5	2.5	2	721.62	15.21	58.28	4	2	2			50	32	32		
*Rallus striatus*	153.75	7	2	113.16	12.16	58.28	1		1			51		29		
*Vanellus vanellus*	315.75	2	4	961.58	36.32	39.62	10				10	52				25
*Chlidonias hybrida*	151.5	3	2	812.72	29.61	29.69	1	1				53	21			

The Mantel test showed that the annual and seasonal waterbird composition and fragment variables were not spatially autocorrelated (Table S4), indicating that the subsequent comparisons of avian composition among fragments were effective and in support of the following nested analyses.

### 3.2 Nestedness of waterbird assemblages among seasons

The community composition of annual, spring, summer, autumn, and winter waterbirds were all significantly nested using the nestedness metric of NeD. The habitats were also significantly nested (Table 4; Figure S1). The same results were obtained using the WNODF nestedness metric, except when using the WNODF_c_ null model test for the summer waterbirds (*p* = .057; Table S5).

**TABLE 4:** Results of the nestedness analyses of 27 lakeside wetland fragments around Lake Dianchi using the NeD program conducted on the species‐by‐site matrix for different seasonal birds and habitat‐by‐site on 27 fragments at Lake Dianchi

Season	Species richness	Metric	Index	*Z*‐score	*p*
Annual	53	NODF	64.09	18.91	<.001
Spring	33	NODF	54.01	8.08	<.001
Summer	34	NODF	72.12	14.29	<.001
Autumn	40	NODF	50.13	9.19	<.001
Winter	28	NODF	62.05	15.00	<.001
Habitat		NODF	49.22	2.30	<.05

### 3.3 Mechanisms determining nestedness

The nestedness of annual and all four seasonal communities of waterbirds was not determined by passive sampling. There were only three, four, one, four, and five observations for annual, spring, summer, autumn, and winter waterbirds, respectively, which lay within ±1 *SD* of the expected species–area curve computed from the random placement model (Figure S2).

The PCA of the five fragment habitat variables provided two principal components (PCs) with eigenvalues higher than one. PC1 was interpreted as an index of fragments with larger areas and higher habitat diversity. PC2 was interpreted as an index of fragments experiencing disturbance and isolation from the nearest larger fragment (Table 5; Figure S3).

**TABLE 5:** Principal component analysis (PCA) of the fragment habitat variables

	Annual, Spring, Summer, Autumn					Winter				
	PC1	PC2	PC3	PC4	PC5	PC1	PC2	PC3	PC4	PC5
Importance										
Eigenvalue	2.3	1.54	0.56	0.36	0.24	2.16	1.55	0.64	0.41	0.24
Proportion explained	0.46	0.31	0.11	0.07	0.05	0.43	0.31	0.13	0.08	0.05
Cumulative proportion	0.46	0.77	0.88	0.95	1	0.43	0.74	0.87	0.95	1
Variable scores										
Area (ln‐transformed)	−1.36	0.13	−0.29	−0.09	−0.56	−1.35	0.29	−0.01	−0.09	−0.55
Habitat diversity	−1.29	0.36	−0.39	−0.32	0.47	−1.24	0.47	−0.51	0.21	0.47
Anthropogenic disturbance	0.72	1.04	−0.74	0.36	−0.02	0.83	0.82	−0.66	−0.55	−0.02
Isolation 1	0.58	1.22	0.33	−0.57	−0.12	0.74	1.11	0.04	0.69	−0.12
Isolation 2	0.92	−0.9	−0.61	−0.5	−0.07	0.57	−1.07	−0.83	0.34	−0.09

Note

The table shows the eigenvalues, percentage of variance explained by each component, and scores of each variable in the component.

The rank orders of fragments for the annual and seasonal waterbirds were not significantly related to anthropogenic disturbance (Table 6); thus, anthropogenic disturbance contributed minimally to the observed nestedness. The rank orders for annual and seasonal waterbirds were significantly related to both the area and the habitat diversity of each fragment and correlated with species traits linked to extinction tendencies (i.e., occupied habitat and area requirement; Table 6). In particular, species occupying less habitat (high habitat specificity) and requiring large areas were subsets of species with low habitat specificity and small area requirements. Thus, selective extinction and habitat nestedness were the leading causes of waterbird nestedness in the lakeside fragments at Dianchi. The fragment ranks (rearranging, whose higher rank represents higher species diversity) of the annual, spring, summer, and winter waterbirds were significantly positively associated with Isolation 1 (Table 6). Thus, the farther the distance to the nearest larger fragment, the more species were observed. However, the ranks of each fragment for the annual, autumn, and winter waterbirds were negatively correlated with Isolation 2 (distance to the species pool). Thus, with an increase in the distance to the species pool, a reduction in species richness was observed. Therefore, the selective colonization hypothesis could explain the observed nestedness of the annual and seasonal waterbirds.

**TABLE 6:** Partial Spearman rank correlations between rank orders of fragments and species using the online NeD program and orders of fragments and species after rearranging the matrix according to each explanatory variable

Season	Habitat variables					Species ecological traits					
	Fragment area (ha)	Habitat diversity	Human disturbance	Isolation 1 (m)	Isolation 2 (m)	Body size (mm)	Clutch size (*n*)	Occupied habitat (*n*)	Geographic range size (km^2^)	Dispersal ratio (db)	Minimum area requirement (ha)
Annual	0.62**	0.60**	−0.21	0.42*	−0.46*	0.10	0.13	0.73**	0.09	0.02	−0.81**
Spring	0.63**	0.57**	−0.19	0.40*	−0.31	0.01	−0.09	0.62**	−0.07	0.19	−0.76**
Summer	0.69**	0.66**	−0.23	0.51**	−0.37	0.23	0.17	0.72**	−0.16	0.17	−0.74**
Autumn	0.56**	0.58**	−0.13	0.29	−0.43*	0.05	0.08	0.80**	0.03	0.02	−0.78**
Winter	0.56**	0.49*	−0.27	0.59**	−0.50**	−0.20	−0.15	0.57**	0.05	0.06	−0.68**

Note

Isolation 1, the distance of each fragment to the nearest larger fragment; Isolation 2, the distance of each fragment to the pool fragment. S10 was the species pool for annual, spring, summer, and autumn waterbirds, and S06 was the species pool for wintering waterbirds. Bold values indicate significant results.

*p < .05; **p < .01.

## 4 DISCUSSIONS

Nested analyses play an important role in conservation biology and are vital tools for explaining the causes of the nestedness of community composition on islands or fragments that resemble island habitats. In this study, we found that nestedness was present in annual and seasonal waterbird assemblages in plateau lakeside fragments, further proving that nestedness is a typical pattern in biotas across different habitats. Selective extinction and habitat nestedness are two common mechanisms that can explain nestedness (Ulrich et al., 2009), and we found that selective colonization could also explain the observed nestedness in this study.

The passive sampling hypothesis is a prerequisite for other hypothesis tests (Wang et al., 2013). In this study, we found that regardless of annual or seasonal surveys, passive sampling played a minor role in the development of nestedness. This result agreed with previous studies on similar freshwater (Li et al., 2019; McAbendroth et al., 2005) and woodlot (Wang et al., 2013) habitats and implied that environmental variables might be the key factors that lead to avian nestedness.

The area of the fragment is a pivotal factor affecting the diversity of different seasonal waterbirds. There may be two reasons for this: (a) More suitable habitats may be supplied by a larger area, which coincides with the traditional theory of area–species (Wiens, 1992); the basic definition of nestedness is that species assembly on small islands is a subset of species assembly on the nearest larger island (Cutler, 1994). (b) As with the hypothesis of selective extinction, the species with large area requirements may be sensitive to the landscape area and have no choice but to choose the larger habitat (Patterson, 1990). The area is essential to most taxa (Schouten et al., 2007; Wang et al., 2010), and in the present study, we provided further proof that wetland waterbird assemblages are affected by the fragment area (Benassi et al., 2007).

Habitat diversity was positively correlated with the species diversity of the fragments. This finding may have been obtained because different waterbirds require different habitats (Ma et al., 2010); thus, greater habitat heterogeneity may attract a variety of waterbirds by supplying standing, foraging, and sheltering environments (Ma et al., 2010). Our findings further demonstrate the commonly occurring pattern of high plant species richness leading to richer avifauna (Wang et al., 2020), while elucidating the critical role of habitat diversity in the small island effect (Chen et al., 2020).

In the current study, we found that the isolations among fragments could contribute to nestedness. Although the significance differed among seasons, they all displayed the same pattern: The farther the distance to the nearest larger fragment, the more species could be observed; the farther the distance to the species pool, the fewer species richness were observed. According to Darlington (1957), the island or fragment with a larger area serves as a species pool for adjacent fragments. Similarly, the fragment occupied by the largest number of species can also be used as a species pool. However, the former assumes that larger fragments hold more species and provide colonization opportunities for species to surround fragments. The latter directly provides species with the opportunity to colonize surrounding fragments. In many cases, the results of these two methods have been inconsistent. This phenomenon has also been observed in other studies, such as those conducted by Xu et al. (2017) and Tan et al. (2020). We also found that ecological traits directly related to species migration ability did not significantly contribute to the observed nested patterns, indicating that isolation degree may not be a clear indicator of the migration ability of a species; alternatively, ecology functions could be obscure (Wang et al., 2013). Fahrig (2013) suggested that isolation degree has a spatial effect, suggesting the latter has a more direct impact and is a relatively accurate method. Therefore, we used the latter to test the selective colonization hypothesis and found that the waterbird compositions in a year and during autumn and winter met the selective colonization hypothesis. The reason for this finding may be that the compositions of waterbirds across the different seasons differed at Dianchi (Wang and Yang, 2020), with distinct migration activities (Henry and Cumming, 2017). During spring and summer, habitats of resident waterbirds predominantly remained unchanged and they showed less movement among fragments; during autumn and winter, mostly migratory birds were observed, who displayed more movement among fragments. This speculation requires further investigation, such as individual‐based tracking research.

Anthropogenic disturbance is a critical cause of nestedness (Fernández‐Juricic, 2002). Examples of such disturbance include traffic (Fernández‐Juricic and Jokimäki, 2001; Platt and Lill, 2006) and noise (González‐Oreja et al., 2012). However, not all nestedness is affected by anthropogenic disturbance. Murgui (2010) found that anthropogenic disturbance did not significantly affect the distribution of birds in Valencia. Murgui (2010) also suggested that birds sensitive to anthropogenic disturbance could choose larger habitats, while birds in small areas are not always sensitive to anthropogenic disturbance. Therefore, the effect of anthropogenic disturbance may be negligible in small landscape areas where most species can tolerate such disturbance. In the present study, we found that seasonal waterbirds were not sensitive to anthropogenic disturbance. There may be two explanations for this: (a) Most of these disturbances (pedestrians, angling, and traffic) occurred at the edge of the wetland patch, and the strength of disturbance is possibly weakened in fragment areas that are large enough to enable birds to avoid threats (Fox and Madsen, 1997; Martín et al., 2015). (b) The factors that decide the habitat choices of birds are complex and interlinked (Coetzee and Chown, 2016; Ma et al., 2010), and birds may have no option but to select a habitat that is suitable in all aspects, including enduring severe disturbance (Cody, 1987). Habitat filtering may be the most important process controlling the structure of waterbird communities in the Yunnan–Guizhou Plateau wetlands. This statement is supported by the observation that most migrants were gathered in the temporary mudflats produced by constructing facilities for lakeside parks at Dianchi, as there were no suitable natural habitats.

In the present study, we found that fragments with larger areas and higher aquatic vegetation heterogeneity contained more waterbirds; however, this remains an indecisive guide for wetland managers when setting the least area criterion of constructed wetlands. Therefore, combined with the requirement for stormwater runoff (Malaviya and Singh, 2012) or pollution abatement efficiency (Nivala et al., 2018), wetland construction must integrate more ecosystem services (Kim et al., 2011). For improved waterbird conservation, we suggest that future studies should aim to quantify the appropriate wetland area, according to waterbird composition characteristics and their life history, to determine the smallest area necessary for constructing artificial wetlands in given regions (Garrett‐Walker et al., 2020). Moreover, vegetation configuration, which affects waterbird composition (Wang et al., 2020), usually varies with ecological succession. Plants with a competitive advantage, such as invasive species, may homogenize wetland habitats by excluding other species (Zhang, Wen, et al., 2020). Notably, cost‐effective methods for maintaining or improving high habitat heterogeneity, such as water level regulation, should be explored (Lin et al., 2016; Zhang et al., 2019).

## 5 CONCLUSIONS

The mechanisms underlying nestedness are complex, intertwined, and dynamic. In the present study, we found that area and vegetation type were key factors driving the nestedness of waterbirds irrespective of the waterbird type (annual, spring, summer, autumn, or winter waterbirds). Therefore, we suggest that constructed wetland landscape areas and habitat heterogeneity should be as large and high as possible, respectively, to provide sufficient habitat diversity for various waterbirds. To better implement wetland construction and management, further research should be conducted to determine the smallest area necessary for constructing artificial wetlands based on the composition of wetland species and their life history. Furthermore, we warrant further research on the configuration and succession of aquatic vegetation to maintain high habitat heterogeneity for the conservation of various waterbirds.

## CONFLICT OF INTEREST

The authors declare no competing interests.

## AUTHOR CONTRIBUTION

**Rongxing Wang:** Conceptualization (supporting); Data curation (lead); Formal analysis (lead); Funding acquisition (supporting); Investigation (lead); Methodology (lead); Writing‐original draft (lead); Writing‐review & editing (lead). **Xiaojun Yang:** Conceptualization (lead); Data curation (supporting); Formal analysis (supporting); Funding acquisition (lead).

## Supporting information

Fig S1‐S3

Table S1‐S5

## Data Availability

The data used for analyses provided in the article are available at Dryad under https://doi.org/10.5061/dryad.w0vt4b8pt
