# Supplementary material for: Nestedness theory suggests wetland fragments with large areas and macrophyte diversity benefit waterbirds
Source: Ecol Evol. 2021 Aug 16;11(18):12651–64. doi: 10.1002/ece3.8009 (PMC8462146; doi:10.1002/ece3.8009)
Supplement: Supplementary file 1 — Fig S1‐S3 [file ECE3-11-12651-s001.pdf]

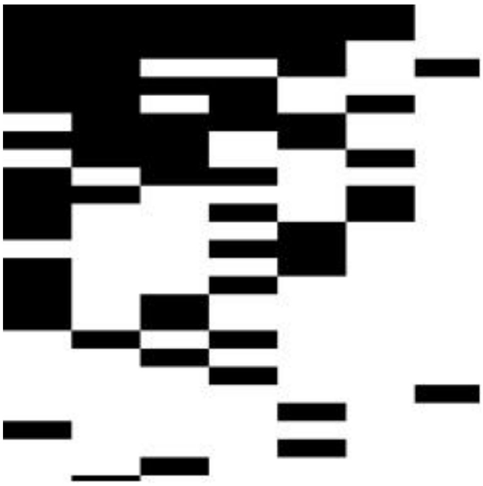

(a) Habitat packed matrix

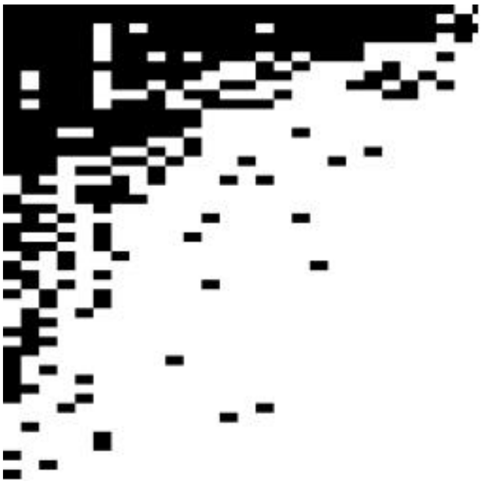

(b) Annual packed matrix

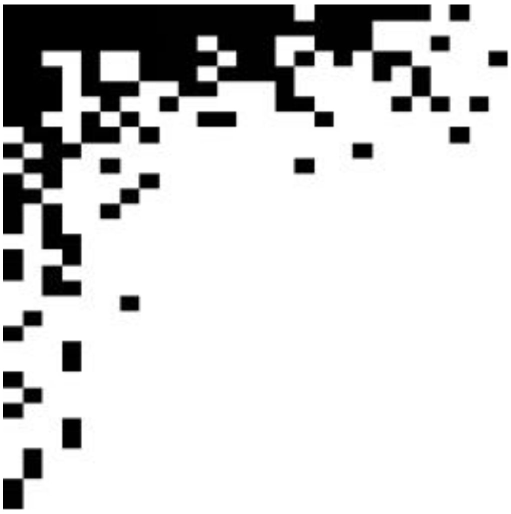

(c) Spring packed matrix

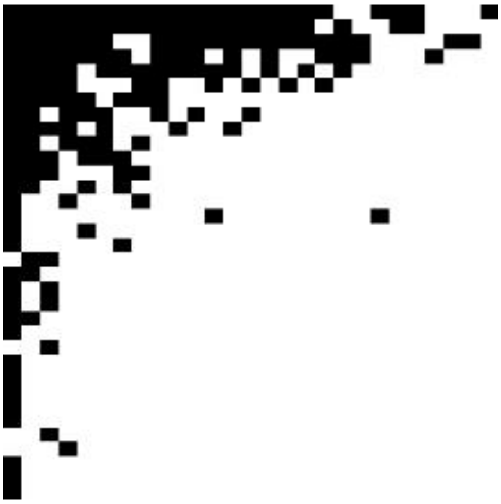

(d) Summer packed matrix

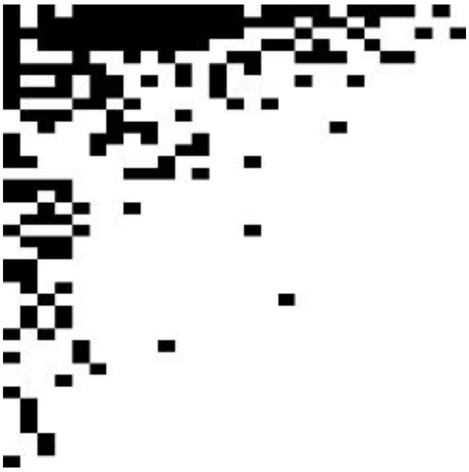

(e) Autumn packed matrix

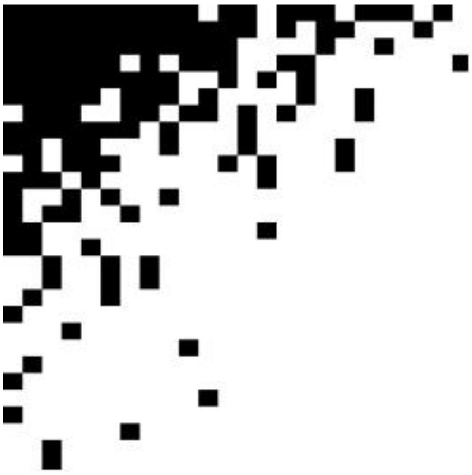

(f) W inter packed matrix

Fig S1 Maximally packed matrix graphic examined using the NeD program. The Vertical represent the fragments order from higher species to low species, the horizontal represent the species.

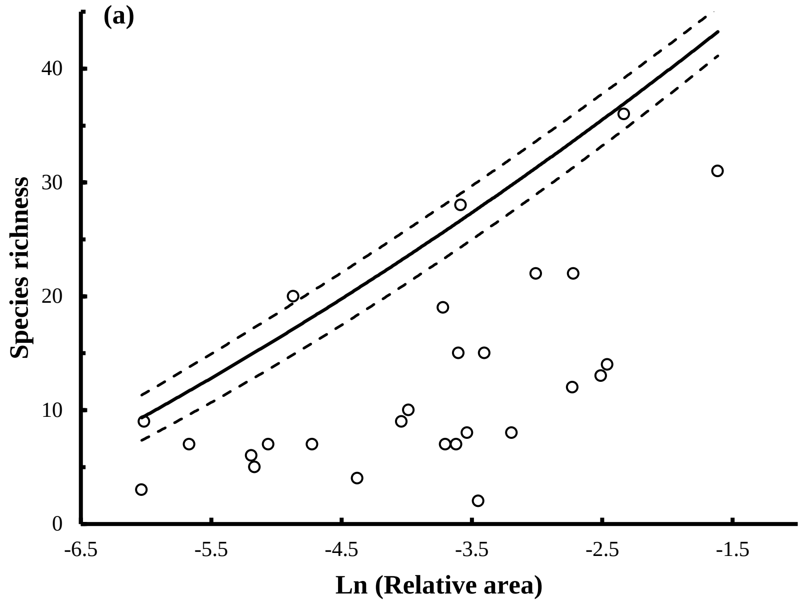
(a)
40
30
20
10
0
-6.5
-5.5
-4.5
-3.5
-2.5
-1.5
Species richness
Ln (Relative area)

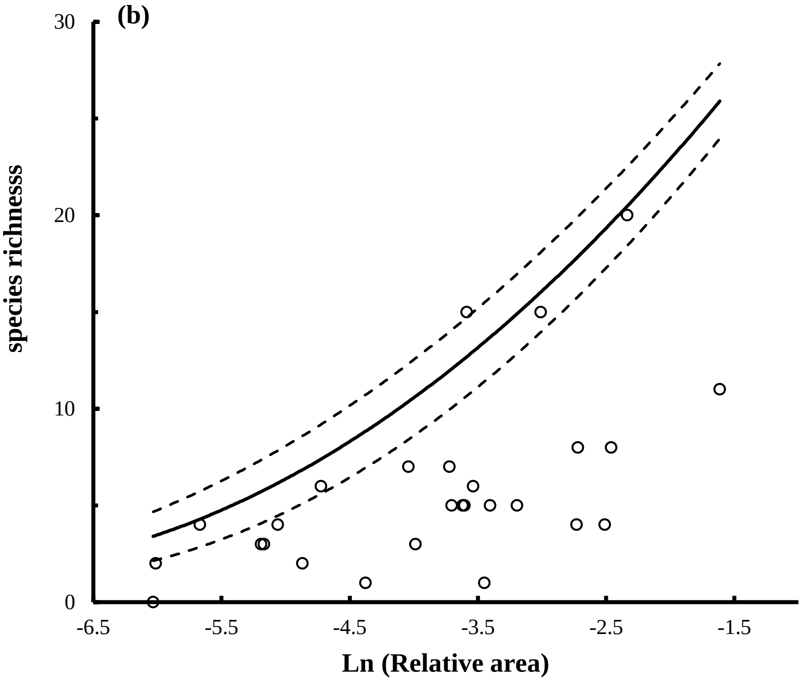
(b)
30
20
10
0
-6.5
-5.5
-4.5
-3.5
-2.5
-1.5
species richnesss
Ln (Relative area)

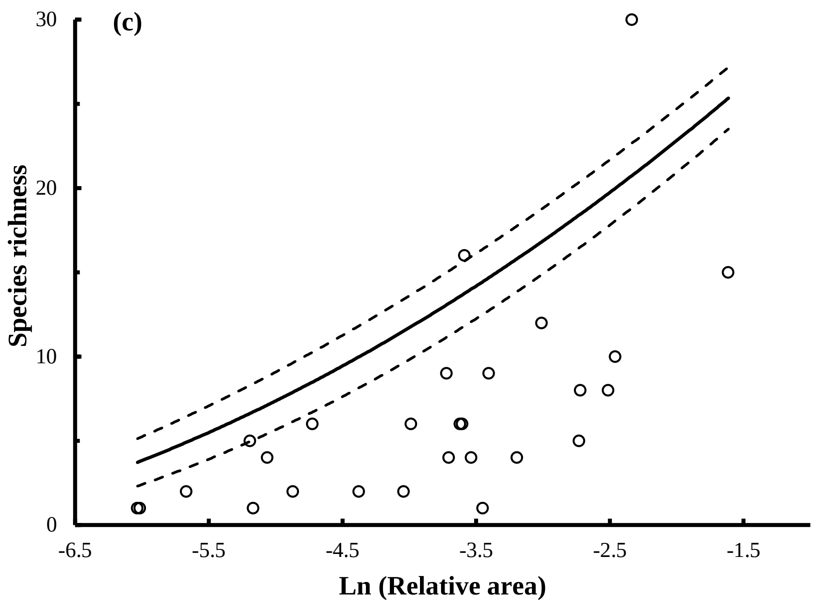
(c)
Species richness
30
20
10
0
-6.5
-5.5
-4.5
-3.5
-2.5
-1.5
Ln (Relative area)

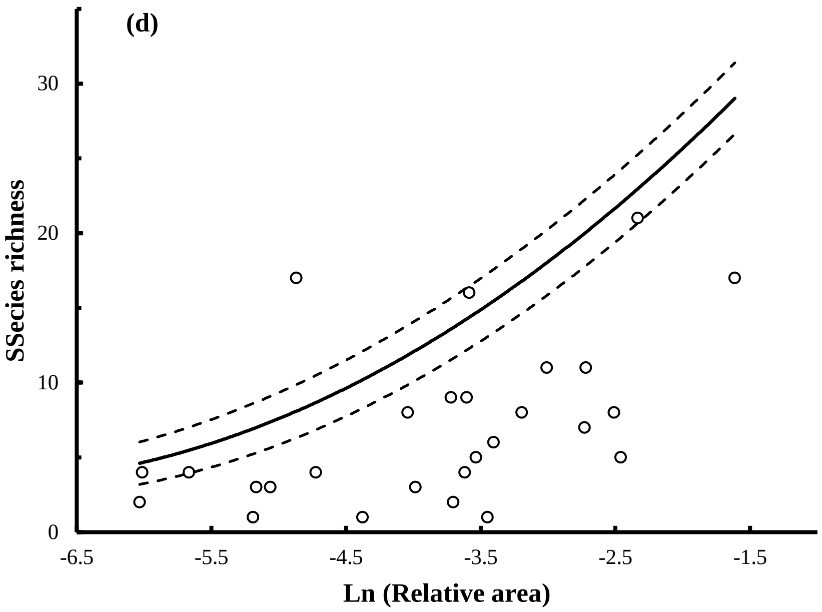
(d)
SSecies richness
30
20
10
0
-6.5
-5.5
-4.5
-3.5
-2.5
-1.5
Ln (Relative area)

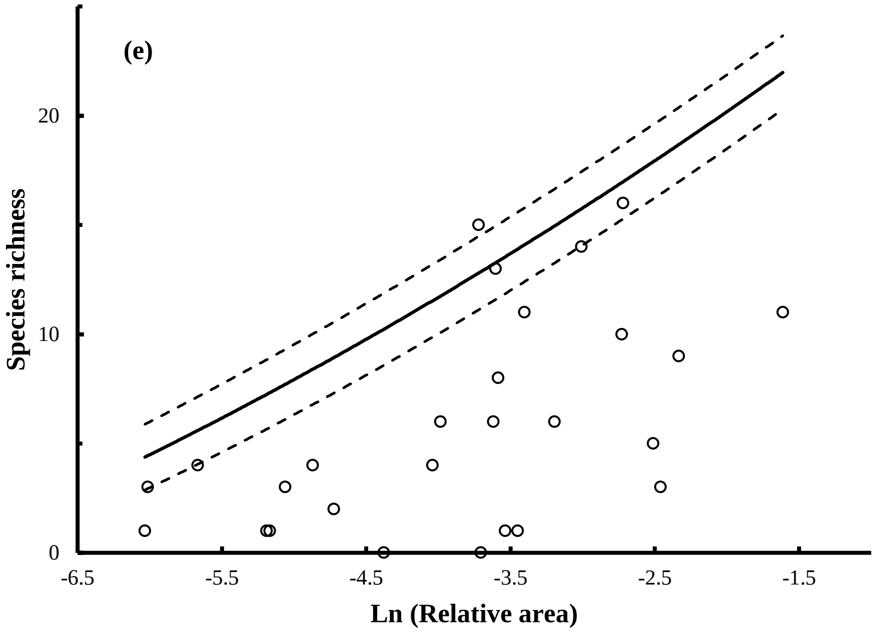

Fig S2 Comparison of observed data to expected values under the random placement models for (a) annual waterbirds, (b) spring waterbirds, (c) summer waterbirds, (d) autumn waterbirds, and (d) winter waterbirds on 27 27 lakeside wetland fragments around Lake Dianchi, Yunnan, China. Expected values (solid line) and associated standard deviations (±1 SD; dashed lines) are shown. Open circles represent observed species richness.

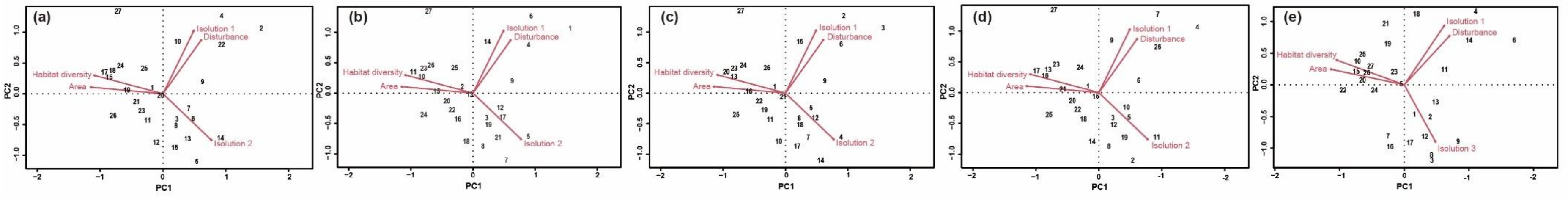

Fig S3 Principal component analyses (PCAs) of fragment variables under the rank order in annual (a), spring (b), summer (c), autumn (d), and winter (d) surveys.
