# Supplementary material for: Nestedness theory suggests wetland fragments with large areas and macrophyte diversity benefit waterbirds
Source: Ecol Evol. 2021 Aug 16;11(18):12651–64. doi: 10.1002/ece3.8009 (PMC8462146; doi:10.1002/ece3.8009)
Supplement: Supplementary file 2 — Table S1‐S5 [file ECE3-11-12651-s002.docx]

| **Table S1** Characteristics of 27 lakeside wetland fragments around Lake Dianchi, China. | | | | | | | | | | | | | | | | | | | | | | | | | | | |
| --- | --- | --- | --- | --- | --- | --- | --- | --- | --- | --- | --- | --- | --- | --- | --- | --- | --- | --- | --- | --- | --- | --- | --- | --- | --- | --- | --- |
| **Fragment code** | **S01** | **S02** | **S03** | **S04** | **S05** | **S06** | **S07** | **S08** | **S09** | **S10** | **s11** | **S12** | **S13** | **S14** | **S15** | **S16** | **S17** | **S18** | **S19** | **S20** | **S21** | **S22** | **S23** | **S24** | **S25** | **S26** | **S27** |
| Area (hectares) | 119.90 | 3.81 | 19.98 | 14.57 | 16.13 | 39.62 | 16.67 | 29.69 | 51.41 | 58.28 | 39.24 | 48.83 | 16.38 | 19.09 | 2.08 | 3.43 | 4.62 | 1.44 | 3.35 | 1.47 | 17.52 | 24.66 | 14.80 | 11.17 | 10.58 | 5.34 | 7.55 |
| HE (hectares) | 26.15 | 3.30 | 10.69 | 8.55 | 12.85 | 3.11 | 0.00 | 12.21 | 19.53 | 8.85 | 6.94 | 8.14 | 2.70 | 0.00 | 0.00 | 0.00 | 0.00 | 0.00 | 3.35 | 0.00 | 10.38 | 0.00 | 0.00 | 10.63 | 0.00 | 3.99 | 5.29 |
| LE (hectares) | 0.00 | 0.00 | 0.00 | 1.29 | 0.16 | 0.00 | 5.95 | 1.12 | 9.79 | 0.75 | 0.00 | 14.13 | 4.93 | 0.00 | 0.00 | 3.43 | 0.00 | 0.00 | 0.00 | 0.00 | 0.00 | 0.00 | 0.00 | 0.00 | 0.00 | 0.00 | 2.26 |
| HF (hectares) | 0.00 | 0.00 | 0.00 | 1.59 | 0.00 | 0.00 | 8.36 | 5.75 | 22.08 | 20.50 | 6.61 | 22.09 | 0.00 | 16.96 | 2.08 | 0.00 | 0.00 | 0.00 | 0.00 | 0.00 | 7.14 | 5.66 | 14.80 | 0.54 | 0.00 | 0.00 | 0.00 |
| LF (hectares) | 0.00 | 0.00 | 0.00 | 2.02 | 1.41 | 0.00 | 0.00 | 0.12 | 0.00 | 0.00 | 0.00 | 0.00 | 8.75 | 0.00 | 0.00 | 0.00 | 0.00 | 0.00 | 0.00 | 0.00 | 0.00 | 6.09 | 0.00 | 0.00 | 0.00 | 0.41 | 0.00 |
| Mudflat (hectares) | 71.86 | 0.00 | 0.00 | 0.00 | 0.00 | 0.00 | 0.00 | 0.00 | 0.00 | 0.00 | 0.00 | 0.00 | 0.00 | 0.00 | 0.00 | 0.00 | 4.62 | 0.00 | 0.00 | 0.00 | 0.00 | 0.00 | 0.00 | 0.00 | 0.00 | 0.00 | 0.00 |
| Mixed (hectares) | 5.61 | 0.51 | 9.29 | 0.60 | 0.00 | 36.51 | 2.15 | 0.45 | 0.00 | 18.29 | 2.97 | 0.00 | 0.00 | 0.00 | 0.00 | 0.00 | 0.00 | 1.44 | 0.00 | 1.47 | 0.00 | 0.00 | 0.00 | 0.00 | 0.00 | 0.00 | 0.00 |
| Pond (hectares) | 16.28 | 0.00 | 0.00 | 0.52 | 1.71 | 0.00 | 0.21 | 10.04 | 0.00 | 9.90 | 22.73 | 4.46 | 0.00 | 2.13 | 0.00 | 0.00 | 0.00 | 0.00 | 0.00 | 0.00 | 0.00 | 12.91 | 0.00 | 0.00 | 10.58 | 0.95 | 0.00 |
| SHDI | 1.05 | 0.39 | 0.69 | 1.29 | 0.68 | 0.28 | 1.03 | 1.26 | 1.05 | 1.37 | 1.12 | 1.24 | 0.99 | 0.35 | 0.00 | 0.00 | 0.00 | 0.00 | 0.00 | 0.00 | 0.68 | 1.02 | 0.00 | 0.19 | 0.00 | 0.72 | 0.61 |
| Isolation 1 (km) | 2.60 | 2.60 | 4.39 | 4.39 | 6.58 | 2.70 | 2.70 | 4.18 | 5.49 | 5.49 | 4.53 | 2.33 | 3.16 | 3.08 | 3.83 | 11.02 | 11.02 | 14.02 | 0.30 | 1.70 | 1.41 | 1.41 | 1.12 | 1.17 | 0.40 | 2.30 | 1.72 |
| Isolation 2 (km, for annul, spring,  summer, autumn waterbirds) | 28.91 | 28.66 | 28.41 | 25.98 | 21.83 | 15.56 | 13.78 | 9.09 | 5.49 | 0.00 | 4.53 | 5.46 | 8.62 | 11.28 | 12.06 | 14.20 | 25.18 | 39.18 | 38.15 | 38.13 | 38.03 | 37.00 | 36.01 | 35.00 | 35.00 | 35.07 | 37.11 |
| Isolation 2 (km, for winter waterbirds) | 17.42 | 16.06 | 14.22 | 10.89 | 6.58 | 0.00 | 2.70 | 6.56 | 10.24 | 15.56 | 16.47 | 14.50 | 14.51 | 13.50 | 9.85 | 9.11 | 16.12 | 28.91 | 28.05 | 28.13 | 28.66 | 28.08 | 26.96 | 26.28 | 26.44 | 25.56 | 27.26 |
| Number of habitats | 1 | 4 | 2 | 1 | 4 | 1 | 3 | 2 | 1 | 4 | 1 | 2 | 1 | 2 | 1 | 4 | 3 | 4 | 1 | 2 | 1 | 2 | 3 | 1 | 2 | 2 | 2 |
| Annual observed richness(n) | 31 | 7 | 15 | 19 | 7 | 22 | 28 | 22 | 14 | 36 | 11 | 13 | 15 | 2 | 7 | 5 | 20 | 3 | 6 | 9 | 8 | 8 | 7 | 10 | 9 | 4 | 7 |
| Annual expected richness Chao1 | 51.23 | 7.98 | 15.13 | 36.97 | 7.50 | 22.67 | 31.00 | 25.12 | 26.41 | 38.00 | 12.00 | 15.24 | 15.25 | 2.00 | 7.98 | 5.23 | 22.98 | 3.00 | 6.96 | 16.77 | 8.49 | 8.50 | 7.00 | 12.22 | 9.00 | 6.67 | 7.00 |
| Annual expected richness Chao1(SE) | 20.17 | 2.20 | 0.44 | 23.58 | 1.32 | 1.30 | 3.24 | 3.65 | 17.02 | 2.64 | 2.27 | 3.38 | 0.73 | 0.34 | 2.19 | 0.69 | 3.22 | 0.39 | 2.15 | 11.34 | 1.31 | 1.32 | 0.54 | 3.35 | 0.26 | 3.88 | 0.37 |
| Annual survey completeness | 0.99 | 0.97 | 1.00 | 0.99 | 1.00 | 1.00 | 0.99 | 0.99 | 0.96 | 1.00 | 1.00 | 0.99 | 1.00 | 1.00 | 0.96 | 0.95 | 0.96 | 1.00 | 0.93 | 0.89 | 0.99 | 1.00 | 1.00 | 0.96 | 1.00 | 0.70 | 1.00 |
| Annual nestedness matrix rank | 2 | 19 | 8 | 7 | 18 | 5 | 3 | 4 | 10 | 1 | 12 | 11 | 9 | 27 | 21 | 24 | 6 | 26 | 23 | 14 | 16 | 17 | 22 | 13 | 15 | 25 | 20 |
| Spring observed richness(n) | 11 | 4 | 5 | 7 | 5 | 8 | 15 | 15 | 8 | 20 | 4 | 4 | 5 | 1 | 4 | 3 | 2 | 0 | 3 | 2 | 6 | 5 | 5 | 3 | 7 | 1 | 6 |
| Spring expected richness Chao1 | 11.99 | 4.95 | 7.97 | 7.00 | 5.00 | 8.25 | 16.00 | 18.98 | 12.41 | 21.33 | 4.00 | 4.00 | 7.93 | 1.00 | 5.88 | 4.50 | 2.00 |  | 3.00 | 2.00 | 6.25 | 5.00 | 5.00 | 3.80 | 7.50 | 1.00 | 6.24 |
| Spring expected richness Chao1(SE) | 2.25 | 2.06 | 4.34 | 0.50 | 0.40 | 0.73 | 1.86 | 5.27 | 7.05 | 1.84 | 0.58 | 0.26 | 4.29 | 0.28 | 3.52 | 2.87 | 0.47 |  | 0.46 | 0.46 | 0.72 | 0.37 | 0.50 | 1.73 | 1.31 | 0.31 | 0.72 |
| Spring survey completeness | 0.98 | 0.91 | 0.97 | 1.00 | 1.00 | 1.00 | 0.99 | 0.98 | 0.94 | 0.99 | 1.00 | 1.00 | 0.93 | 1.00 | 0.88 | 0.63 | 1.00 |  | 1.00 | 1.00 | 0.98 | 1.00 | 1.00 | 0.73 | 0.99 | 1.00 | 0.98 |
| Spring nestedness matrix rank | 4 | 19 | 15 | 8 | 14 | 6 | 3 | 2 | 5 | 1 | 18 | 17 | 13 | 26 | 16 | 22 | 24 | 27 | 21 | 23 | 10 | 12 | 11 | 20 | 7 | 25 | 9 |
| Summer observed richness(n) | 15 | 4 | 9 | 9 | 6 | 8 | 16 | 12 | 10 | 30 | 5 | 8 | 6 | 1 | 2 | 1 | 2 | 1 | 5 | 1 | 4 | 4 | 4 | 6 | 2 | 2 | 6 |
| Summer expected richness Chao1 | 21.22 | 4.00 | 9.99 | 11.98 | 6.49 | 8.25 | 25.96 | 19.95 | 22.26 | 33.12 | 5.99 | 8.25 | 6.00 | 1.00 | 2.50 | 1.00 | 2.00 | 1.00 | 6.93 | 1.00 | 4.47 | 4.00 | 4.00 | 10.37 | 2.00 | 2.50 | 6.00 |
| Summer expected richness Chao1(SE) | 7.51 | 0.50 | 1.85 | 4.45 | 1.31 | 0.73 | 10.22 | 11.58 | 16.82 | 3.65 | 2.18 | 0.72 | 0.42 | 0.00 | 1.11 | 0.31 | 0.46 | 0.28 | 2.96 | 0.31 | 1.26 | 0.34 | 0.52 | 7.00 | 0.00 | 1.11 | 0.54 |
| Summer survey completeness | 0.97 | 1.00 | 0.97 | 0.98 | 0.99 | 0.99 | 0.98 | 0.97 | 0.90 | 0.99 | 0.99 | 0.98 | 1.00 | 1.00 | 0.67 | 1.00 | 1.00 | 1.00 | 0.65 | 1.00 | 0.95 | 1.00 | 1.00 | 0.92 | 1.00 | 0.67 | 1.00 |
| Summer nestedness matrix rank | 3 | 19 | 7 | 6 | 13 | 9 | 2 | 4 | 5 | 1 | 15 | 8 | 12 | 27 | 23 | 26 | 22 | 25 | 14 | 24 | 18 | 17 | 16 | 11 | 21 | 20 | 10 |
| Autumn observed richness(n) | 17 | 3 | 6 | 9 | 4 | 11 | 16 | 11 | 5 | 21 | 7 | 8 | 9 | 1 | 4 | 3 | 17 | 2 | 1 | 4 | 5 | 8 | 2 | 3 | 8 | 1 | 4 |
| Autumn expected richness Chao1 | 21.49 | 3.00 | 6.00 | 9.99 | 4.00 | 11.99 | 17.59 | 11.17 | 5.95 | 22.14 | 7.50 | 8.99 | 9.99 | 1.00 | 4.91 | 3.43 | 23.08 | 2.00 | 1.00 | 4.00 | 5.89 | 9.00 | 2.50 | 3.46 | 8.00 | 1.00 | 4.97 |
| Autumn expected richness Chao1(SE) | 4.79 | 0.25 | 0.48 | 1.86 | 0.53 | 1.86 | 2.15 | 0.53 | 2.10 | 1.62 | 1.32 | 1.85 | 1.85 | 0.28 | 1.99 | 1.16 | 6.03 | 0.46 | 0.31 | 0.36 | 1.69 | 2.24 | 1.11 | 1.23 | 0.59 | 0.05 | 2.10 |
| Autumn survey completeness | 0.99 | 1.00 | 1.00 | 0.98 | 1.00 | 0.98 | 0.97 | 0.99 | 0.91 | 0.99 | 0.99 | 0.98 | 0.98 | 1.00 | 0.85 | 0.89 | 0.95 | 1.00 | 1.00 | 1.00 | 0.82 | 0.99 | 0.67 | 0.93 | 1.00 | 1.00 | 0.94 |
| Autumn nestedness matrix rank | 3 | 22 | 13 | 8 | 19 | 6 | 4 | 5 | 15 | 1 | 12 | 11 | 7 | 27 | 18 | 21 | 2 | 24 | 26 | 17 | 14 | 10 | 23 | 20 | 9 | 25 | 16 |
| Winter observed richness(n) | 12 | 4 | 12 | 16 | 7 | 17 | 9 | 15 | 4 | 10 | 11 | 6 | 14 | 2 | 5 | 2 | 5 | 2 | 2 | 4 | 2 | 7 | 0 | 7 | 5 | 0 | 3 |
| Winter expected richness Chao1 | 17.99 | 4.00 | 14.00 | 33.93 | 7.99 | 17.50 | 9.00 | 22.98 | 4.00 | 11.00 | 13.99 | 6.99 | 15.99 | 2.00 | 6.93 | 2.00 | 5.00 | 2.00 | 2.00 | 4.90 | 2.00 | 7.50 |  | 11.38 | 5.00 |  | 3.00 |
| Winter expected richness Chao1(SE) | 7.16 | 0.24 | 3.74 | 23.53 | 2.22 | 1.03 | 0.54 | 11.63 | 0.48 | 1.86 | 4.50 | 2.20 | 3.73 | 0.28 | 3.62 | 0.18 | 0.57 | 0.40 | 0.07 | 1.97 | 0.40 | 1.32 |  | 7.00 | 0.34 |  | 0.34 |
| Winter survey completeness | 0.99 | 1.00 | 1.00 | 0.98 | 0.99 | 0.99 | 1.00 | 0.99 | 1.00 | 0.99 | 0.99 | 0.98 | 0.99 | 1.00 | 0.93 | 1.00 | 1.00 | 1.00 | 1.00 | 0.84 | 1.00 | 1.00 |  | 0.92 | 1.00 |  | 1.00 |
| Winter nestedness matrix rank | 6 | 17 | 5 | 2 | 10 | 1 | 9 | 3 | 18 | 7 | 8 | 13 | 4 | 23 | 15 | 24 | 14 | 22 | 25 | 19 | 21 | 12 | 26 | 11 | 16 | 26 | 20 |
| HE, High emerging plant area; LE, Low emerging plant area; HF, High floating plant area; LF, Low floating plant area; Mixed, Mixed vegetation area; SHDI, Shannon -Wiener Diversity Index of habitats. | | | | | | | | | | | | | | | | | | | | | | | | | | | |

| **Table S2** Distances (km) among 27 lakeside wetland fragments around Lake Dianchi, China. | | | | | | | | | | | | | | | | | | | | | | | | | | |
| --- | --- | --- | --- | --- | --- | --- | --- | --- | --- | --- | --- | --- | --- | --- | --- | --- | --- | --- | --- | --- | --- | --- | --- | --- | --- | --- |
|  | **S01** | **S02** | **S03** | **S04** | **S05** | **S06** | **S07** | **S08** | **S09** | **S10** | **S11** | **S12** | **S13** | **S14** | **S15** | **S16** | **S17** | **S18** | **S19** | **S20** | **S21** | **S22** | **S23** | **S24** | **S25** | **S26** |
| S02 | 2.60 |  |  |  |  |  |  |  |  |  |  |  |  |  |  |  |  |  |  |  |  |  |  |  |  |  |
| S03 | 6.98 | 4.38 |  |  |  |  |  |  |  |  |  |  |  |  |  |  |  |  |  |  |  |  |  |  |  |  |
| S04 | 10.71 | 8.28 | 4.39 |  |  |  |  |  |  |  |  |  |  |  |  |  |  |  |  |  |  |  |  |  |  |  |
| S05 | 12.78 | 10.81 | 8.00 | 4.31 |  |  |  |  |  |  |  |  |  |  |  |  |  |  |  |  |  |  |  |  |  |  |
| S06 | 17.42 | 16.07 | 14.22 | 10.89 | 6.58 |  |  |  |  |  |  |  |  |  |  |  |  |  |  |  |  |  |  |  |  |  |
| S07 | 17.04 | 16.05 | 14.93 | 12.21 | 8.07 | 2.70 |  |  |  |  |  |  |  |  |  |  |  |  |  |  |  |  |  |  |  |  |
| S08 | 21.05 | 20.36 | 19.58 | 16.92 | 12.74 | 6.56 | 4.72 |  |  |  |  |  |  |  |  |  |  |  |  |  |  |  |  |  |  |  |
| S09 | 25.13 | 24.52 | 23.73 | 20.94 | 16.70 | 10.24 | 8.81 | 4.18 |  |  |  |  |  |  |  |  |  |  |  |  |  |  |  |  |  |  |
| S10 | 28.91 | 28.66 | 28.41 | 25.98 | 21.83 | 15.56 | 1.38 | 9.09 | 5.49 |  |  |  |  |  |  |  |  |  |  |  |  |  |  |  |  |  |
| S11 | 27.15 | 27.29 | 27.74 | 25.96 | 22.10 | 16.47 | 14.17 | 10.03 | 8.00 | 4.53 |  |  |  |  |  |  |  |  |  |  |  |  |  |  |  |  |
| S12 | 24.85. | 24.96 | 25.43 | 23.71 | 19.92 | 14.50 | 12.09 | 8.25 | 7.09 | 5.46 | 2.33 |  |  |  |  |  |  |  |  |  |  |  |  |  |  |  |
| S13 | 22.54 | 22.91 | 23.83 | 22.61 | 19.15 | 14.51 | 11.88 | 9.06 | 9.26 | 8.62 | 5.00 | 3.16 |  |  |  |  |  |  |  |  |  |  |  |  |  |  |
| S14 | 19.57 | 20.06 | 21.26 | 20.42 | 17.28 | 13.50 | 10.80 | 9.21 | 10.70 | 11.28 | 8.05 | 6.01 | 3.08 |  |  |  |  |  |  |  |  |  |  |  |  |  |
| S15 | 17.09 | 17.19 | 17.90 | 16.73 | 13.46 | 9.85 | 7.17 | 6.79 | 9.62 | 12.06 | 10.11 | 7.78 | 5.95 | 3.83 |  |  |  |  |  |  |  |  |  |  |  |  |
| S16 | 14.80 | 14.86 | 15.63 | 14.68 | 11.69 | 9.11 | 6.62 | 7.782 | 11.22 | 14.20 | 12.44 | 10.11 | 8.21 | 5.75 | 2.33 |  |  |  |  |  |  |  |  |  |  |  |
| S17 | 5.16 | 6.90 | 10.50 | 12.93 | 13.36 | 16.12 | 14.97 | 18.22 | 22.06 | 25.18 | 22.91 | 20.66 | 18.10 | 15.04 | 13.12 | 11.02 |  |  |  |  |  |  |  |  |  |  |
| S18 | 11.51 | 13.33 | 17.03 | 21.36 | 24.12 | 28.91 | 28.36 | 32.06 | 36.01 | 39.18 | 36.71 | 34.53 | 31.78 | 2.87 | 27.12 | 25.04 | 14.02 |  |  |  |  |  |  |  |  |  |
| S19 | 10.70 | 12.63 | 16.46 | 20.75 | 23.39 | 28.05 | 27.44 | 31.09 | 35.02 | 38.15 | 35.65 | 33.47 | 30.72 | 27.64 | 26.09 | 24.02 | 13.01 | 1.08 |  |  |  |  |  |  |  |  |
| S20 | 10.81 | 12.78 | 16.65 | 20.92 | 23.52 | 28.13 | 27.50 | 31.12 | 35.03 | 38.13 | 35.60 | 33.423 | 30.65 | 27.57 | 26.07 | 24.01 | 13.00 | 1.22 | 0.30 |  |  |  |  |  |  |  |
| S21 | 11.56 | 13.72 | 17.76 | 21.96 | 24.34 | 28.66 | 27.87 | 31.31 | 35.15 | 38.03 | 35.33 | 33.20 | 30.35 | 27.28 | 26.00 | 24.02 | 13.10 | 2.60 | 2 | 1.70 |  |  |  |  |  |  |
| S22 | 11.28 | 13.59 | 17.77 | 21.86 | 24.02 | 28.08 | 27.17 | 30.48 | 34.26 | 37.00 | 34.21 | 32.11 | 29.22 | 26.16 | 25.01 | 23.09 | 12.28 | 3.94 | 3.16 | 2.88 | 1.41 |  |  |  |  |  |
| S23 | 10.18 | 12.52 | 16.73 | 20.79 | 22.91 | 26.96 | 26.07 | 29.41 | 33.21 | 36.01 | 33.28 | 31.15 | 28.30 | 25.23 | 24.00 | 22.05 | 11.20 | 4.17 | 3.20 | 2.97 | 2.06 | 1.12 |  |  |  |  |
| S24 | 9.83 | 12.27 | 16.56 | 20.52 | 22.44 | 26.28 | 25.30 | 28.54 | 32.29 | 35.00 | 32.21 | 30.10 | 27.23 | 24.16 | 23.02 | 21.10 | 10.36 | 5.32 | 4.31 | 4.10 | 3.20 | 2.00 | 1.17 |  |  |  |
| S25 | 10.11 | 12.57 | 16.88 | 20.81 | 22.68 | 26.44 | 25.43 | 28.61 | 32.34 | 35.00 | 32.17 | 30.07 | 27.18 | 24.12 | 23.04 | 21.15 | 10.47 | 5.59 | 4.61 | 4.39 | 3.35 | 2.06 | 1.41 | 0.40 |  |  |
| S26 | 8.60 | 10.90 | 15.90 | 19.17 | 21.36 | 25.56 | 24.76 | 28.23 | 32.09 | 35.07 | 32.49 | 30.32 | 27.53 | 24.46 | 23.02 | 21.00 | 10.03 | 4.31 | 3.23 | 3.13 | 3.10 | 2.69 | 1.64 | 1.90 | 2.30 |  |
| S27 | 10.02 | 12.09 | 16.08 | 20.30 | 22.78 | 27.26 | 26.58 | 30.15 | 34.05 | 37.11 | 34.56 | 32.39 | 29.61 | 26.53 | 25.05 | 23.00 | 12.00 | 2.24 | 1.17 | 1.04 | 1.72 | 2.40 | 2.15 | 3.20 | 3.52 | 2.09 |

| **Table S3** Ecological traits of waterbirds, accumulated individuals, and rank orders by NeD program in annual, spring, summer, autumn, and winter seasons between March 2013 to February 2014 in the 27 lakeside wetland fragments around Lake Dianchi, China. | | | | | | | | | | | | | | | |
| --- | --- | --- | --- | --- | --- | --- | --- | --- | --- | --- | --- | --- | --- | --- | --- |
| **Species** | **Body size (mm)** | **Clutch size (n)** | **Habitat specificity** | **Geographic range size (km^2^)** | **Dispersal ratio (dp)** | **Accumulated individuals** | | | | | **Nested matrix rank** | | | | |
|  |  |  |  |  |  | **Annual** | **Spring** | **Summer** | **Autumn** | **Winter** | **Annual** | **Spring** | **Summer** | **Autumn** | **Winter** |
| *Egretta garzetta* | 596.5 | 2.5 | 8 | 295.09 | 35.1 | 1736 | 214 | 678 | 453 | 391 | 1 | 2 | 1 | 3 | 1 |
| *Gallinula chloropus* | 190 | 8 | 2 | 961.58 | 12.18 | 2829 | 887 | 781 | 768 | 393 | 2 | 1 | 2 | 2 | 2 |
| *Amaurornis phoenicurus* | 302 | 6 | 7 | 526.51 | 17.27 | 138 | 44 | 48 | 20 | 26 | 3 | 4 | 3 | 5 | 7 |
| *Ardeola bacchus* | 262.15 | 3 | 5 | 908.62 | 32.01 | 458 | 76 | 15 | 269 | 98 | 4 | 5 | 9 | 1 | 4 |
| *Podiceps ruficollis* | 158.15 | 5.5 | 2 | 961.58 | 18.92 | 1289 | 347 | 326 | 317 | 299 | 5 | 3 | 5 | 4 | 3 |
| *Ixobrychus sinensis* | 331.5 | 7 | 1 | 606.69 | 18.91 | 54 | 3 | 41 | 8 | 2 | 6 | 27 | 4 | 9 | 18 |
| *Larus ridibundus* | 386.75 | 3 | 1 | 961.58 | 25.68 | 1190 | 109 | 7 | 118 | 956 | 7 | 8 | 20 | 15 | 5 |
| *Bubulcus ibis* | 509.75 | 6 | 5 | 955.92 | 33.99 | 690 | 232 | 81 | 155 | 222 | 8 | 6 | 10 | 7 | 9 |
| *Porzana fusca* | 115.75 | 7 | 6 | 600.05 | 15.86 | 55 | 16 | 32 | 7 |  | 9 | 7 | 8 | 20 |  |
| *Egretta intermedia* | 666.5 | 2 | 6 | 291.12 | 39.68 | 81 | 10 | 57 | 14 |  | 10 | 15 | 6 | 13 |  |
| *Ardea cinerea* | 888 | 5 | 6 | 961.58 | 38.32 | 170 | 7 | 29 | 47 | 87 | 11 | 13 | 15 | 12 | 6 |
| *Tringa glareola* | 110 | 2 | 7 | 961.58 | 31.06 | 143 | 14 | 98 | 23 | 8 | 12 | 12 | 7 | 8 | 12 |
| *Capella gallinago* | 171.5 | 2 | 9 | 961.58 | 15.08 | 130 | 16 | 3 | 43 | 68 | 13 | 9 | 17 | 6 | 8 |
| *Tringa hypoleucos* | 189.15 | 2.5 | 5 | 961.58 | 19.52 | 30 |  | 8 | 10 | 12 | 14 |  | 13 | 14 | 10 |
| *Charadrius dubius* | 168 | 3.5 | 8 | 961.58 | 32.53 | 63 | 9 | 47 |  | 7 | 15 | 19 | 12 |  | 11 |
| *Vanellus cinereus* | 321 | 2 | 6 | 676.36 | 35.57 | 281 | 23 |  | 234 | 24 | 16 | 11 |  | 10 | 13 |
| *Ixobrychus cinnamomeus* | 329.5 | 2.5 | 6 | 360.9 | 17.59 | 24 | 8 | 11 | 5 |  | 17 | 10 | 11 | 26 |  |
| *Tringa ochropus* | 132 | 3.5 | 1 | 961.58 | 31.12 | 40 | 23 | 8 | 7 | 2 | 18 | 14 | 34 | 21 | 20 |
| *Nycticorax nycticorax* | 515 | 2 | 6 | 821.36 | 31.92 | 44 |  |  | 42 | 2 | 19 |  |  | 11 | 22 |
| *Charadrius alexandrinus* | 161.5 | 2 | 7 | 873.52 | 31.08 | 16 |  | 1 | 9 | 6 | 20 |  | 30 | 27 | 14 |
| *Capella stenura* | 151.15 | 2 | 3 | 961.58 | 17.31 | 11 |  | 3 | 3 | 5 | 21 |  | 25 | 23 | 15 |
| *Pluvialis fulva* | 121.15 | 2.5 | 7 | 961.58 | 31.86 | 7 |  | 3 | 4 |  | 22 |  | 19 | 19 |  |
| *Fulica atra* | 391 | 9 | 10 | 961.58 | 12.86 | 118 | 29 | 1 | 11 | 77 | 23 | 30 | 31 | 30 | 16 |
| *Himantopus himantopus* | 353.75 | 2 | 2 | 961.58 | 21.02 | 224 | 10 | 187 | 27 |  | 24 | 29 | 22 | 16 |  |
| *Charadrius placidus* | 110.75 | 3.5 | 6 | 796.58 | 32.05 | 10 |  | 7 | 3 |  | 25 |  | 14 | 24 |  |
| *Charadrius mongolus* | 188.5 | 3 | 6 | 612.69 | 33.11 | 11 | 7 |  | 4 |  | 26 | 17 |  | 25 |  |
| *Anas crecca* | 388.5 | 9.5 | 7 | 961.58 | 16.18 | 52 | 1 |  | 10 | 41 | 27 | 26 |  | 18 | 27 |
| *Anas poecilorhyncha* | 570.5 | 9.5 | 7 | 961.58 | 15.91 | 58 | 9 | 32 | 16 | 1 | 28 | 33 | 33 | 31 | 21 |
| *Calidris ferruginea* | 110 | 2 | 1 | 905.97 | 31.51 | 18 | 5 | 6 | 7 |  | 29 | 24 | 28 | 35 |  |
| *Anas strepera* | 299.5 | 10 | 6 | 961.58 | 17.18 | 146 | 19 |  |  | 127 | 30 | 31 |  |  | 17 |
| *Calidris subminuta* | 153.15 | 2 | 6 | 961.58 | 18.81 | 18 |  | 11 | 7 |  | 31 |  | 26 | 17 |  |
| *Calidris alpina* | 195.5 | 2 | 2 | 631.37 | 19.2 | 2 |  |  | 2 |  | 32 |  |  | 28 |  |
| *Tadorna ferruginea* | 592 | 9 | 11 | 959.02 | 31.57 | 21 |  |  | 5 | 16 | 33 |  |  | 39 | 23 |
| *Numenius phaeopus* | 217.15 | 2 | 6 | 887.38 | 31.11 | 6 |  | 1 | 5 |  | 34 |  | 24 | 22 |  |
| *Tringa erythropus* | 193 | 2 | 5 | 961.58 | 30.35 | 3 |  |  | 3 |  | 35 |  |  | 29 |  |
| *Glareola maldivarum* | 131.5 | 3 | 6 | 658.72 | 21.21 | 106 | 42 | 64 |  |  | 36 | 16 | 18 |  |  |
| *Tringa totanus* | 170 | 2 | 3 | 860.71 | 30.81 | 7 | 4 | 3 |  |  | 37 | 28 | 21 |  |  |
| *Porzana pusilla* | 172.15 | 7.5 | 5 | 838.81 | 12.62 | 4 | 1 | 2 | 1 |  | 38 | 22 | 16 | 34 |  |
| *Anas platyrhynchos* | 523.75 | 9 | 6 | 961.58 | 16.78 | 5 | 4 |  |  | 1 | 39 | 18 |  |  | 26 |
| *Hydrophasianus chirurgus* | 225 | 2 | 2 | 191.11 | 37.1 | 4 | 1 |  |  | 3 | 40 | 25 |  |  | 19 |
| *Calidris minuta* | 140 | 3.5 | 3 |  | 32.97 | 4 | 1 | 3 |  |  | 41 | 23 | 27 |  |  |
| *Ixobrychus eurhythmus* | 335.5 | 2 | 3 | 623.35 | 17.68 | 2 |  | 1 | 1 |  | 42 |  | 23 | 32 |  |
| *Larus brunnicephalus* | 221 | 3 | 5 | 629.57 | 21.03 | 13 | 12 |  |  | 1 | 43 | 20 |  |  | 28 |
| *Botaurus stellaris* | 676.75 | 5 | 7 | 771.69 | 31.6 | 1 |  |  |  | 1 | 44 |  |  |  | 24 |
| *Arenaria interpres* | 115.15 | 2 | 2 | 857.37 | 31.51 | 1 |  |  | 1 |  | 45 |  |  | 38 |  |
| *Calidris ruficollis* | 156.15 | 2 | 6 | 961.58 | 31.5 | 2 |  |  | 2 |  | 46 |  |  | 36 |  |
| *Recurvirostra avosetta* | 217.75 | 2 | 6 | 959.02 | 31.78 | 1 |  |  | 1 |  | 47 |  |  | 37 |  |
| *Ardea purpurea* | 901.5 | 2.5 | 5 | 606.69 | 36.2 | 1 |  |  | 1 |  | 48 |  |  | 40 |  |
| *Anas acuta* | 567.5 | 8.5 | 7 | 961.58 | 18.15 | 2 |  |  | 2 |  | 49 |  |  | 33 |  |
| *Rostratula benghalensis* | 150.5 | 2.5 | 1 | 721.62 | 15.21 | 4 | 2 | 2 |  |  | 50 | 32 | 32 |  |  |
| *Rallus striatus* | 153.75 | 7 | 8 | 113.16 | 12.16 | 1 |  | 1 |  |  | 51 |  | 29 |  |  |
| *Vanellus vanellus* | 315.75 | 2 | 6 | 961.58 | 36.32 | 10 |  |  |  | 10 | 52 |  |  |  | 25 |
| *Chlidonias hybrida* | 151.5 | 3 | 8 | 812.72 | 29.61 | 1 | 1 |  |  |  | 53 | 21 |  |  |  |

| **Table S4** Mantel test of annual and different seasonal waterbird composition and habitat variables on 27 lakeside wetland fragments aroung Lake Dianchi, China. | | |
| --- | --- | --- |
|  | **R** | ***p*** |
| Annual bird composition | 0.08 | 0.07 |
| Spring bird composition | -0.04 | 0.78 |
| Summer bird composition | 0.03 | 0.26 |
| Autumn bird composition | -0.04 | 0.82 |
| Winter bird composition | 0.04 | 0.22 |
| Area (log_10_–transformed) | 0.07 | 0.12 |
| Habitat diversity | 0.05 | 0.22 |
| Disturbance | -0.34 | 0.77 |

| **Table S5** Results of nestedness analyses using the program NODF conducted on the species-by-sites abundance matrix for waterbird assemblages on 27 lakeside wetlans fragments around Lake Dianchi, China | | | | | | | | | | |
| --- | --- | --- | --- | --- | --- | --- | --- | --- | --- | --- |
| **Seasons** | **Nestedness metric** | **WNODF _obs_** | ***aa* null model** | |  | ***ss* null model** | |  | ***rc* null model** | |
|  |  |  | **WNODF_exp_ (SD)** | ***P*** |  | **WNODF_exp_ (SD)** | ***P*** |  | **WNODF_exp_ (SD)** | ***P*** |
| Annual | WNODF | 41.84 | 59.56(2.36) | <0.0001 |  | 72.53(3.78) | <0.0001 |  | 58.42(2.20) | <0.0001 |
|  | WNODFc | 51.10 | 73.30(1.53) | <0.0001 |  | 77.22(2.24) | <0.0001 |  | 68.62(2.21) | <0.0001 |
|  | WNODFr | 39.81 | 56.58 | <0.0001 |  | 71.54(4.17) | <0.0001 |  | 56.21(2.36) | <0.0001 |
| Spring | WNODF | 32.15 | 50.32(2.83) | <0.0001 |  | 62.28(6.20) | <0.0001 |  | 48.62(3.04) | <0.0001 |
|  | WNODFc | 35.47 | 57.12(4.21) | <0.0001 |  | 63.55(5.23) | <0.0001 |  | 53.39(4.21) | <0.0001 |
|  | WNODFr | 29.95 | 45.90(3.40) | <0.0001 |  | 61.56(7.30) | <0.0001 |  | 45.55(3.14) | <0.0001 |
| Summer | WNODF | 44.46 | 54.21(3.37) | 0.0018 |  | 67.07(5.78) | 0.0000 |  | 52.78(3.38) | 0.0068 |
|  | WNODFc | 46.39 | 61.34(5.31) | 0.0024 |  | 70.16(6.55) | 0.0001 |  | 58.19(4.89) | 0.0077 |
|  | WNODFr | 43.26 | 49.86(4.18) | 0.0571 |  | 65.26(5.83) | 0.0001 |  | 49.49(3.49) | 0.0368 |
| Autumn | WNODF | 32.12 | 49.92(2.53) | <0.0001 |  | 62.16(6.18) | <0.0001 |  | 47.90(2.59) | <0.0001 |
|  | WNODFc | 41.92 | 62.92(4.29) | <0.0001 |  | 69.39(5.20) | <0.0001 |  | 57.81(4.32) | 0.0001 |
|  | WNODFr | 27.70 | 44.14(2.80) | <0.0001 |  | 58.99(6.99) | <0.0001 |  | 43.51(2.34) | <0.0001 |
| Winter | WNODF | 34.95 | 57.50(2.95) | <0.0001 |  | 66.87(4.77) | <0.0001 |  | 54.84(3.08) | <0.0001 |
|  | WNODFc | 38.07 | 58.59(3.33) | <0.0001 |  | 63.15(4.29) | <0.0001 |  | 54.23(3.18) | <0.0001 |
|  | WNODFr | 32.25 | 56.69(4.33) | <0.0001 |  | 70.24(5.59) | <0.0001 |  | 55.50(3.73) | <0.0001 |
